# Identifying the clinical and histopathological characteristics of amelanotic melanoma: a case series

**DOI:** 10.1093/omcr/omae029

**Published:** 2024-04-25

**Authors:** Aroon Sohail, Svetlana Kavaklieva

**Affiliations:** Lancaster Medical School, Lancaster University, Lancaster, United Kingdom; Royal Lancaster Infirmary, University Hospitals of Morecambe Bay NHS Foundation Trust, Lancaster, United Kingdom; Department of Dermatology, Royal Lancaster Infirmary, University Hospitals of Morecambe Bay NHS Foundation Trust, Lancaster, United Kingdom

## Abstract

Amelanotic melanoma (AM) is a subtype of melanoma where the lesion demonstrates no pigmentation. This can lead to delays in referral with studies showing a higher mortality rate. To determine the characteristics of AM lesions, we conducted a retrospective analysis of patients with confirmed AM. Of the 16 patients, 68.75% were male and the mean age at diagnosis was 78 years. The most common location for AM was the head (37.5%) which also demonstrated a higher mitotic rate (10.67 mm^2^) compared to the average (7.31 mm^2^). More than half of the lesions (56%) had been present for more than 1 year. With a misdiagnosis rate of 87.5%, the likelihood of delays were evident. There was no unifying feature on clinical assessment, however conspicuous vessel findings were noted on 62.5% of lesions. We have demonstrated that AM continues to remain a missed diagnosis with the potential for a more lethal cancer to form.

## INTRODUCTION

Amelanotic melanoma (AM) is a subtype of melanoma where the lesion phenotype demonstrates little to no pigmentation. It accounts for less than 2% of melanoma diagnosis, but is associated with a higher mortality rate [1, 2]. Amelanotic melanoma remains a diagnostic challenge as it mimics various benign and malignant conditions. A misdiagnosis rate of up to 89% has been reported [3]. It is often detected at later stages in disease progression, potentially secondary to delays in referral due to misdiagnosis with a consequently more lethal disease developing. Thomas et al. conducted an international population based study which highlighted several key characteristics regarding AM [4]. The study identified 8% of melanomas were histopathologically amelanotic (275 out of 3467). Amelanotic melanoma was identified to generally have a higher tumour stage at diagnosis in comparison to pigmented melanoma. Furthermore, AM had a greater hazard of death compared to pigmented melanoma, with a hazard ratio of 2.0. The study concluded that survival after diagnosis of AM was poorer than pigmented melanoma, due to a more advanced stage at diagnosis, likely due to difficulties and delays in diagnosis. There are few reports in the current literature regarding AM, which limits our understanding of its associated characteristics. Herein we present a case series of 16 patients with AM, reporting the clinical and histological features of the disease. The objective of this report is to educate and increase the awareness of AM by increasing the understanding of the clinical and histopathological characteristics associated with AM.

## REPORT

In a 10-year period at a single centre in the Northwest of England, patients with a histological diagnosis of AM were enrolled and data collated retrospectively. Consent for research was obtained from each patient at biopsy.

Table 1 summarises demographics, clinical presentation and histological characteristics of the lesions. A total of 16 patients were diagnosed with AM. There were 11 men and 5 women with a mean age at diagnosis of 78 years (male mean age 77 years, female mean age 79 years). Amelanotic melanoma was most commonly seen to manifest in the head (37.5%), followed by the legs (25%), back (18.8%), arms (12.5%) and neck (6.25%). The average size of the lesion at diagnosis was 13.88 mm (minimum size 5 mm, maximum size 35 mm). The average Breslow thickness was 3.16 mm (minimum thickness 0.2 mm, maximum thickness 5.7 mm) and an average mitotic rate of 7.31 mm^2^ (minimum rate of 0 mm^2^, maximum rate of 23 mm^2^). Interestingly, the average Breslow thickness of lesions on the back (4.07 mm) was notably higher than the overall average. In contrast, the average mitotic rate was highest in lesions of the head (10.67 mm^2^) and leg (9 mm^2^). The most common histological subtype was nodular (62.5%), followed by superficial spreading (18.75%), lentigo maligna melanoma (6.25%), desmoplastic (6.25%) and balloon (6.25%). Desmoplastic and lentigo maligna melanoma subtypes both had a notably higher Breslow thickness than the average at 5.5 mm.

**Table 1:** Patient demographics and summary of AM features (SSM—superficial spreading melanoma; LMM—lentigo maligna melanoma; NMSC—non-melanoma skin cancer)

Patient no.	Age at diagnosis	Sex	Site	Size (mm)	History of NMSC	Breslow Thickness (mm)	Mitotic Rate (mm^2^)	Histological Subtype
1	77	Male	Back	10	No	3.0	5	Nodular
2	80	Male	Leg	20	No	2.6	12	SSM
3	88	Male	Head	20	Yes	5.5	6	LMM
4	80	Female	Leg	8	No	1.5	5	SSM
5	72	Male	Arm	5	No	1.7	7	Nodular
6	92	Male	Head	12	No	5.7	23	Nodular
7	80	Female	Leg	8	No	2.6	8	Nodular
8	84	Male	Back	17	–	3.7	1	Nodular
9	73	Male	Back	15	No	5.5	2	Desmoplastic
10	87	Male	Head	9	Yes	5.4	16	Nodular
11	83	Male	Arm	11	No	2.7	2	Nodular
12	80	Female	Head	8	No	1.9	4	Nodular
13	33	Male	Neck	35	No	2.5	0	Balloon
14	75	Female	Leg	10	No	3.8	11	Nodular
15	79	Male	Head	20	No	0.2	0	SSM
16	80	Female	Head	14	No	2.3	15	Nodular

Table 2 summarises the clinical findings and dermoscopic descriptions during clinical examination. In our series, the clinical misdiagnosis rate was 87.5% with squamous cell carcinoma (SCC) and basal cell carcinoma (BCC) the most common misdiagnoses, accounting for 9 of 16 cases. Onset of lesions were an almost even split, with 56% of lesions reported as longstanding (>12 months) and 44% recent (<12 months). Lesions were noted as non-ulcerated (75%), erythematous (50%) or yellow plaque (12.5%), however there was variation amongst the lesions with no unifying common feature. Dermoscopic findings were variable, but conspicuous vessels were noted in 62.5% of lesions. The most common vessel patterns were linear and irregular vessels, both identified in 18.75% of cases with 12.5% of cases showing arborising vessels.

**Table 2:** Summary of AM clinical and dermoscopic findings (BCC—basal cell carcinoma; SCC—squamous cell carcinoma; AM—amelanotic melanoma)

Patient No.	Clinical Description	Dermoscopic Description	Ulceration Present	Clinical Diagnosis	Duration
1	Pearly erythematous nodule	White scar-like centre and enlarged linear tortuous vessels, asymmetric at the periphery	No	BCC	Longstanding
2	Erythematous firm nodule	Crystalline structures, irregular vessels with background inflammation	No	AM	Longstanding
3	Nodular	Irregular vessels, perifollicular rosettes	No	SCC	Longstanding
4	Asymptomatic, ulcerated warty nodule	–	No	SCC	Recent
5	Pink, nodule with surrounding actinic damage	–	No	BCC	Longstanding
6	Bleeding, nodular non-specific	Abnormal enlarged linear vessels	Yes	SCC	Longstanding
7	Bleeding non-healing ulcer	Arborising vessels	Yes	BCC	Recent
8	Erythematous dome shaped nodule	Coiled vessels	No	AM	Recent
9	Erythematous non-tender plaque with dermal involvement	Arborising vessels	No	BCC	Recent
10	Haemorrhagic crust, non-healing	–	No	SCC	Longstanding
11	Bleeding red nodule	Irregular vessels	Yes	SCC	Recent
12	Asymptomatic, erythematous nodule	Well circumscribed enlarged symmetric vessels, no pigment	No	Irritated compound melanocytic naevus	Recent
13	Yellow plaque	Yellow amorphous	No	Xanthelasma	Longstanding
14	Nodular	–	No	Dermatofibroma	Longstanding
15	Erythematous plaque with enlarged pores, attached to previous scar from junctional naevus	–	No	Granuloma faciale	Longstanding
16	Rapidly enlarging yellow nodule, occaisionally bleeds	Linear vessels	Yes	–	Recent

## DISCUSSION

Our case series demonstrates that almost all patients with AM were elderly (>70 years-old). This is in keeping with the general statistics of melanoma incidence which increases with age, with the highest rates in the 85–89 age group for both males and females [5]. Furthermore, our cohort were predominantly male, which also follows the general trend in melanoma incidence where rates are higher in males in the older age groups [5].

The most common site in our series was an exposed area which was the head. This is contrary to the current statistics of melanoma, where the most common location is the trunk [5]. According to the literature, melanomas which develop on the trunk occur more often in the fifth to sixth decades of life, whereas melanomas that develop in high ultra violet (UV) exposed body regions, like the head and neck, occur more commonly in the eighth decade [3]. Due to the older age and mainly exposed sites of involvement, it is most likely that chronic UV exposure rather than intermittent is leading to the development of AM.

The overall Breslow thickness was found to be high, which is in accordance to previous reports and likely to be related to a delay in diagnosis. Furthermore, a higher mitotic rate was noted in AM on the head, which may suggest a more aggressive nature of AM in exposed areas. The most common clinical misdiagnosis of AM was BCC and SCC and this is in agreement with previous reports [6].

Dermoscopic characteristics predominantly consisted of vascular abnormalities. Dermoscopy is a vital component of detecting abnormal features which can suggest AM. Vessel analysis is recommended for lesions which lack pigmentation to identify suspicious lesions, with the 5 + 2 list used to guide vessel analysis [7]. Vascular patterns which raise the suspicion of melanoma include irregular dot, linear irregular, arborising and polymorphic vessels. These patterns have been highlighted in a larger cohort study by Paolino et al. where there was high prevalence of linear looped vessels (58.8%), linear irregular vessels (50.0%) and arborising vessels (47.2%) [8]. Considering 62.5% of the AM lesions in our series had vessel features in accordance with the aforementioned report, we would recommend that any lesion suspicious for AM should have dermoscopy assessment with specific focus on identifying the aforementioned vessel features [9].

We recognise the limitations of our study, principally the small sample size and no follow up. Furthermore, dermoscopic data were often incomplete and lacking detail, making the clinical assessment analysis more difficult. Regardless, we have demonstrated that AM can have a significant misdiagnosis rate with the possibility for a more aggressive cancer to form, potentially leading to unfavourable patient outcomes. We have also demonstrated key features of suspicious lesions which require assessment, notably abnormal vessels. Practitioners in primary and secondary care should be aware of and vigilant in identifying AM and arranging further appropriate investigation.
